# A Colorimetric Sensor for the Highly Selective Detection of Sulfide and 1,4-Dithiothreitol Based on the In Situ Formation of Silver Nanoparticles Using Dopamine

**DOI:** 10.3390/s17030626

**Published:** 2017-03-20

**Authors:** Lingzhi Zhao, Liu Zhao, Yanqing Miao, Chunye Liu, Chenxiao Zhang

**Affiliations:** 1 Department of Pharmacy, Xi’an Medical College, Xi’an 710021, China; miaoyanqing2006@163.com (Y.M.); doris8976@163.com (C.L.); 2 Laboratory of Analytical Chemistry for Life Science of Shaanxi Province, School of Chemistry and Chemical Engineering, Shaanxi Normal University, Xi’an 710062, China; cxzhang@snnu.edu.cn; 3 Beijing Research Center of Agricultural Standards and Testing, Beijing 100097, China; zhaoliu@nercita.org.cn

**Keywords:** sulfide, 1,4-dithiothreitol, silver nanoparticles, colorimetric sensor

## Abstract

Hydrogen sulfide (H_2_S) has attracted attention in biochemical research because it plays an important role in biosystems and has emerged as the third endogenous gaseous signaling compound along with nitric oxide (NO) and carbon monoxide (CO). Since H_2_S is a kind of gaseous molecule, conventional approaches for H_2_S detection are mostly based on the detection of sulfide (S^2−^) for indirectly reflecting H_2_S levels. Hence, there is a need for an accurate and reliable assay capable of determining sulfide in physiological systems. We report here a colorimetric, economic, and green method for sulfide anion detection using in situ formation of silver nanoparticles (AgNPs) using dopamine as a reducing and protecting agent. The changes in the AgNPs absorption response depend linearly on the concentration of Na_2_S in the range from 2 to 15 μM, with a detection limit of 0.03 μM. Meanwhile, the morphological changes in AgNPs in the presence of S^2−^ and thiol compounds were characterized by transmission electron microscopy (TEM). The as-synthetized AgNPs demonstrate high selectivity, free from interference, especially by other thiol compounds such as cysteine and glutathione. Furthermore, the colorimetric sensor developed was applied to the analysis of sulfide in fetal bovine serum and spiked serum samples with good recovery.

## 1. Introduction

Hydrogen sulfide (H_2_S), for centuries considered as a toxic gas with its characteristic odor of rotten egg, displays both acute and chronic toxicity at high concentrations [1,2,3]. On the other hand, recently, H_2_S has emerged as the third endogenous gaseous signaling compound (gasotransmitter) along with nitric oxide (NO) and carbon monoxide (CO) [4,5]. More recent studies have demonstrated that H_2_S is also produced via enzymatic reaction in the brain and several smooth muscles and can mediate a wide range of physiological processes, such as vasodilation, antioxidation, anti-apoptosis, and anti-inflammation [5,6,7,8]. The abnormal level of H_2_S was closely associated with diseases such as Alzheimer’s disease, hypertension, liver cirrhosis, and Down’s syndrome [9,10,11]. Therefore, new methods for the accurate and sensitive detection of H_2_S levels, recognized as providing valuable information on illuminating the physiological roles of H_2_S in biology, are in high demand.

Since H_2_S is a kind of gaseous molecule, conventional approaches for H_2_S detection are mostly based on the detection of sulfide for indirectly reflect H_2_S levels. In aqueous media, sulfide can be found in different forms such as dissolved H_2_S, bisulfide anion (HS^−^, pKa_1_ = 6.88), and sulfide anion (S^2−^, pKa_2_ = 14.15), depending on water pH [12,13]. Thus, there is great importance in developing new and practical methods for sulfide determination to reflect H_2_S levels in biosystems. Several methods have been reported for the determination of sulfide such as electroanalytical and spectrometric methods, high-performance liquid chromatography, and inductively coupled plasma atomic emission spectroscopy (ICP-AES) [14,15,16,17,18,19,20,21,22,23,24]. However, these methods are time-consuming, involve complicated procedures, necessitate expensive and complex equipment, or require specialized skills. Therefore, the further development of highly sensitive, fast-responding, cost-effective, non-polluting, portable devices for monitoring trace levels of sulfide anion in a variety of environmental applications and biosystems is a worthwhile and challenging undertaking.

Owing to the high affinity between many metal ions and sulfide (Ksp of CuS = 1.27 × 10^−36^, Ksp of Ag_2_S = 6.3 × 10^−50^ at room temperature) [25,26], many works have aimed to design a tunable difunctional probe that not only can realize the detection of metal ions but can also be tuned to construct a chemosensing ensemble with metal ions for the detection of S^2−^. Among these metal nanoparticles, colorimetric sensors based on gold nanoparticles (AuNPs) and silver nanoparticles (AgNPs) have attracted much attention because of their unique optical properties. For instance, AgNPs make a strong absorption band that can be observed in the visible to near-UV region of the spectrum, and the color of AgNP suspension depends on the diameter and distance of the nanoparticles. In particular, the dispersed AgNP solution is bright yellow, while the highly aggregated AgNP solution is brownish black. This characteristic leads to new competencies for chemical sensing that are both useful and extraordinarily sensitive for the detection of various species in biological, chemical, and environmental fields. Owing to their simplicity, high sensitivity, and designability, AgNPs based on plasmon resonance have made great achievements in the colorimetric assays for the detection of cysteine, metal ions, and physiologically important species [27,28,29,30,31,32,33,34,35,36,37]. However, the synthesis of AgNPs with distinctive sizes and shapes is usually carried out by chemical reduction with or without stabilizing agents, which is effective but uses many toxic substances, making the process potentially harmful to the environment. Therefore, environmentally friendly or green synthetic methods are in high demand.

**Scheme 1:**
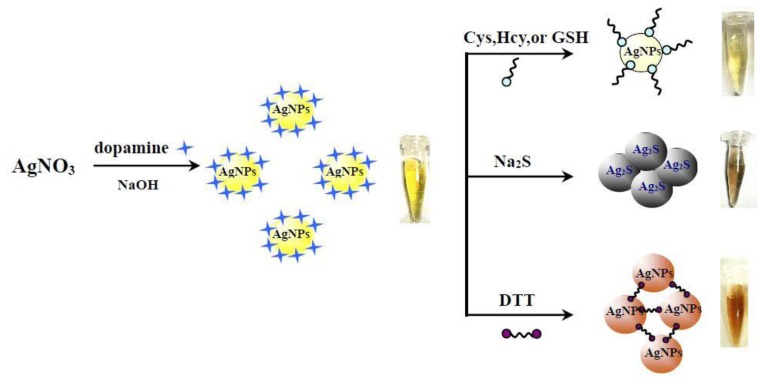
Proposed mechanism of colorimetric determination of S^2−^, DTT, biothiols with silver/dopamine nanoparticles.

In this work, we used silver/dopamine nanoparticles to develop a one-step and colorimetric sensing method for the detection of sulfide. Herein, silver/dopamine nanoparticles were in situ synthesized by using only AgNO_3_, dopamine, NaOH, and Milli-Q water and mixing them in sequence, this process that meets demands for environmentally friendliness or green synthesis. Dopamine could reduce Ag^+^ to AgNPs and functionalize it to form monodispersed AgNPs in alkaline conditions. It is very interesting to note that the addition of sulfide to the initially prepared dopamine-stabilized AgNP colloidal solution decreased the plasmon absorbance of AgNPs at 400 nm and turns the dispersion color from bright yellow to dark brown observed with the naked eye. This phenomenon was elucidated by the degradation of the structure of silver nanoparticles (AgNPs) due to the formation of Ag_2_S by the reaction between Ag^+^ and S^2−^. A graphic representation of the sensor design and the detection strategy is displayed in Scheme 1. Since the AgNPs becomes decomplexed from dopamine, the material properties change, e.g., plasmon absorbance and the yellow color disappear. These features actually form the basis for the development of a technically simple yet effective colorimetric method for the quantitative analysis of sulfide in biosamples. Moreover, considering that thiol compounds have a strong effect on plasmon resonance of AgNPs, we also studied field transmission electron microscope (TEM) images, absorption spectra, and UV-Vis spectra of AgNPs in the presence of different thiol compounds, including L-cysteine (Cys), homocysteine (Hcy), glutathione (GSH), oxidized glutathione (GSSG), 11-mercaptoundecanoic acid (MUA), N-acetyl-L-cysteine (N-Cys), and 1,4-dithiothreitol (DTT). Among these thiol compounds, only DTT, which contained two proximal sulfhydryl groups, can crosslink monodisperse AgNPs to form aggregated AgNPs with a larger particle size and a reddish brown color, resulting in the corresponding changes in the absorbance peak. This feature actually established the development of the technically simple yet effective colorimetric method for the quantitative analysis of DTT, a safe use of this important substance in cell biology, biochemistry, and biomedical applications. In conclusion, a colorimetric sensor for the highly selective detection of sulfide and DTT was developed based on the in situ formation of AgNPs using dopamine, which helps to further establish microfluidic paper-based analytical devices for point-of-use diagnostics without external power supplies or supporting equipment based on the color change of AgNPs.

## 2. Experimental

### 2.1. Reagents and Materials

Dopamine, lactate, glucose, sodium ascorbate, uric acid, 3,4-dihydroxyphenylacetic acid, 5-hydroxytryptamine, and homocysteine (Hcy) were all purchased from Sigma-Aldrich (Shanghai, China). Cysteamine hydrochloride (Cym), L-cysteine (Cys), L-dithiothreitol (DTT), 11-mercaptoundecanoic acid (MUA), glutathione (GSH), oxidized glutathione (GSSG), N-acetyl-L-cysteine (N-Cys) and other amino acids, adenosine 5’-triphosphate disodium salt hydrate (ATP), adenosine 5-diphosphate sodium salt (ADP), adenosine 5-monophosphate monohydrate (AMP), and sodium sulfide non-ahydrate (Na_2_S·9H_2_O) were obtained from the Aladdin company (Shanghai, China). Silver nitrate (AgNO_3_), NaOH, potassium pyrophosphate (PPi), Na_2_SO_4_, NaCl, KNO_3_, Na_2_SO_3_, Na_2_S_2_O_3_, NaH_2_PO_4_, Na_2_HPO_4_, Na_3_PO_4_, Zn(NO_3_)_2_, Fe(NO_3_)_3_, Cu(NO_3_)_2_, CaCl_2_, Mg(Cl)_2_, NaF and other inorganic salts were purchased from Sinopharm Chemical Reagent Co., Ltd. (Shanghai, China). Bovine serum albumin (BSA) was obtained from Shanghai Sangon Biological Engineering Technology and Services Co., Ltd. (Shanghai, China). All other chemicals were at least analytical grade reagents and used without further purification. Aqueous solutions were prepared with Milli-Q water (18.2 M·Ω·cm^−1^). Unless otherwise pointed out, all experiments were carried out at room temperature.

### 2.2. Apparatus

The absorbance and photoluminescence (PL) spectra were recorded in 1 cm quartz cells on a UV-Vis spectrophotometer (UV-2450, Shimadzu Corporation, Tokyo, Japan). Field transmission electron microscope (TEM) studies were carried out with the Tecnai G2 F20 (FEI Company, Hillsboro, OR, USA) operating at a 200 kV accelerated voltage.

### 2.3. Recommended Analytical Procedure

Silver/dopamine nanoparticles were synthesized according to a previously reported method [38]. Twelve microliters of dopamine (4 mM) and 1 mL of ultrapure water were mixed in a 1.5 mL centrifugal tube. Then, 16 μL of NaOH (0.1 M) and 30 μL of AgNO_3_ (8 mM) were added to the tube in sequences. The mixture was incubated at room temperature for 15 min to obtain monodispersed and stable silver/dopamine nanoparticles. For the detection of S^2−^, dithiothreitol, and other analytes, the stock solution (10 mM) of various analytes was first prepared in ultrapure water and then was added into silver/dopamine nanoparticles in the tube at different final concentrations for 20 min before UV-Vis measurements and photographs were taken.

### 2.4. Colorimetric Sensing of S^2−^ in Fetal Bovine Serum

The samples were all prepared by dissolving Na_2_S directly into Milli-Q water as a standard S^2−^ solution. Twenty microliters of the solution at different concentrations and 400 μL of methanol were added to 180 μL of serum and vortexed thoroughly. The mixture was allowed to stand for about 10 min at room temperature to complete protein precipitation. After centrifugation at 13,000 r/min in an Eppendorf centrifuge, the solvent was evaporated to dryness, and samples were redissolved in 20 μL of Milli-Q water as spiked samples. For the colorimetric sensing of S^2−^ in fetal bovine serum, a 20 μL sample was added to 1 mL of the aqueous dispersion of AgNPs. After being allowed to stand for 15 min, the concentrations of S^2−^ in the fetal bovine serum were determined via UV-Vis spectroscopy.

## 3. Results and Discussion

### 3.1. Absorption and TEM Studies of Silver/Dopamine Nanoparticles after Incubation with S^2−^

Because of the degradation of the structure of silver nanoparticles (AgNPs) due to the formation of Ag_2_S by the reaction between Ag^+^ and S^2−^, silver nanoparticles are chosen as an assay material for an effective optical determination of S^2−^. What is more, it is well known that AgNPs display an SPR absorption band at around a wavelength of 400 nm. Since the position of the SPR band depends on the AgNP size and the local environment, such as the pH and the salting strength, AgNP colloidal solution is very sensitive to the addition of analytes with the alteration in SPR spectra and color change in the solution, which can be easily monitored with the naked eye or with a UV-Vis absorption spectrophotometer. In particular, the dispersed AgNP colloidal solution is bright yellow, while the highly aggregated AgNP solution is red or black. From the above, AgNPs serve as an excellent material for employing and developing colorimetric sensors for the detection of physiologically important species in biosystems. Moreover, considering that dopamine has the ability to reduce Ag^+^ to a monodispersed AgNP colloidal solution and functionalize the formed AgNPs in an alkaline solution and at ambient temperature, silver/dopamine nanoparticles, which can obtained by a simple corresponding solution mixed in sequences and does not need complicated synthesis and modification procedures, was chosen in the development of a colorimetric sensor for the detection of S^2−^.

**Figure 1:**
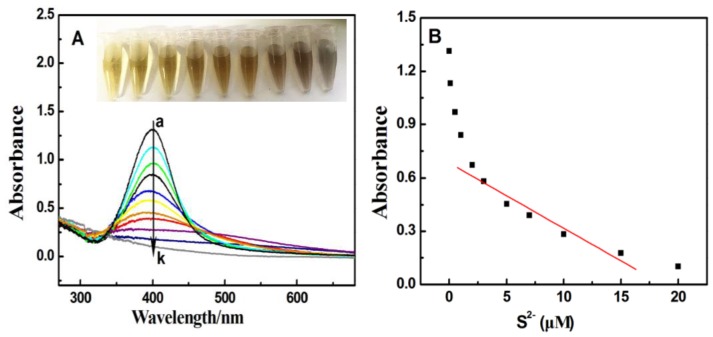
(**A**) UV-Vis absorption responses of AgNPs in the absence (curve a) and presence of different concentrations of S^2−^ (from curve a to curve k: 0, 0.1, 0.5, 1, 2, 3, 5, 7, 10, 15, 20 μM). The color changes of AgNPs before (the first tube) and after the reaction with different concentrations of S^2−^ (from left to right: 0, 0.5, 1, 3, 5, 7, 10, 15, 20 μM) are shown in the inset. The reation time, 20 min. (**B**) UV-Vis absorption responses versus the S^2−^ concentration. The reation time, 20 min.

**Figure 2:**
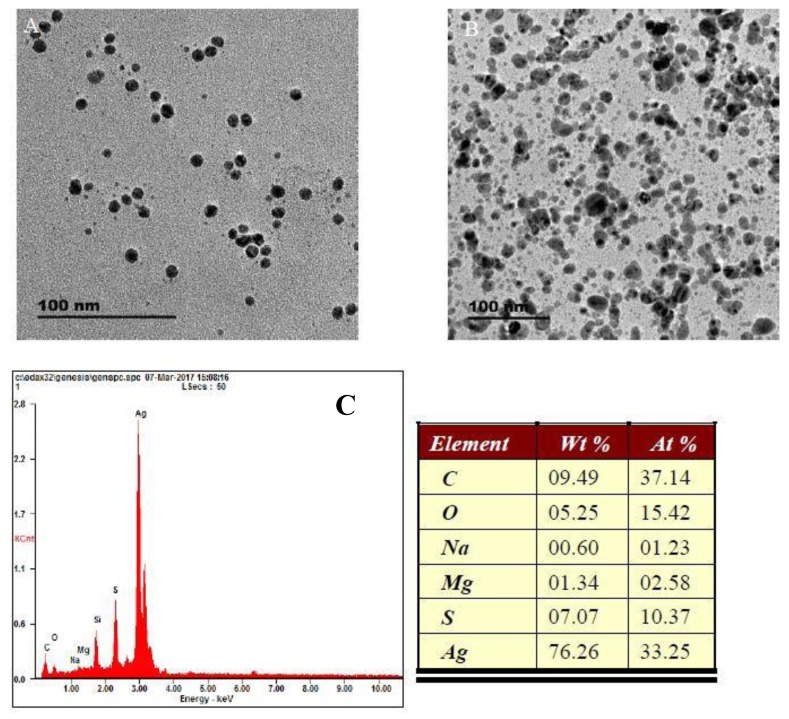
TEM images of dopamine-functionalized AgNPs in the absence (**A**) and the presence (**B**) of 10 μM·S^2−^. The reation time, 20 min. (**C**) Energy disperse X-ray spectroscope (EDS) spectrum of dopamine-functionalized AgNPs in the presence of 10 μM·S^2−^.

To investigate the feasibility of the as-designed sensing method based on the in situ formation of an AgNP colloidal solution using dopamine as a reducing and protecting agent, we conducted UV-Vis absorption spectrum measurements of silver/dopamine nanoparticles in the presence and absence of S^2−^. Curve A of Figure 1A displayed a strong absorbance band centered at about 400 nm with a bright yellow color for silver/dopamine nanoparticles in the absence of S^2−^ (Figure 1A inset: the first tube from left to right), which is the characteristic absorption profile of the as-formed AgNPs. Transmission electron microscopy (TEM) images of the as-formed AgNPs displayed a particle size about 10 nm in diameter, indicated a homogeneous size distribution, and was well dispersed (Figure 2A). The addition of S^2−^ to the initially prepared dopamine-stabilized AgNP colloidal solution sharply decreased the plasmon absorbance of AgNPs at 400 nm and turned the dispersion color from bright yellow (the first tube) to dark brown (Figure 1A inset). Based on the phenomena mentioned above, a possible mechanism was proposed by the degradation of the structure of silver nanoparticles (AgNPs) due to the formation of Ag_2_S, which consequently precipitated from the dispersion by the reaction between Ag^+^ and S^2−^ according to the following equation:

$$4\mathrm{Ag}\;+{\;O}_{2}+\;2H_{2}O\;+\;2{\;S}^{2-}\to 2{\mathrm{Ag}}_{2}S\;+\;4{\mathrm{OH}}^{-}$$

Since the solubility product constant of Ag_2_S is 6.30 × 10^−50^, Ag has a very high reactivity with sulfide in an aqueous solution, which in turn results in the formation of Ag_2_S. In general, the process must be divided into two steps: Figure 1A inset shows the color changes in the AgNPs before and after the reaction, with different concentrations of S^2−^; as can be seen, the color changed from bright yellow (the first tube) to dark brown (Figure 1A inset). The reaction time is 20 min, and the dark brown color can be explained by the fact that the AgNPs became closer to each other and aggregated to some extent after the introduction of S^2−^ in the first 20 min. Another reason is that the as-formed dark colloidal solution must be an Ag_2_S colloidal solution, which was validated by mixing equal amounts of AgNOs and Na_2_S (the product must be Ag_2_S), as shown in Figure S1. We can also obtain the same dark colloidal solution. This is the first step; however, when the reaction time exceeded 1 h, the AgNP colloidal solution was broken down, and the as-formed complex between AgNPs and S^2−^ was consequently precipitated from the dispersion and, after 24 h, this reaction colloidal solution gradually became a transparent dispersion, and small black particles as well as biothiols gathered at the bottom of the tubes. This is the second step. These small black particles must be Ag_2_S. We confirmed the composition of the products in Figure 2C. Ag and S had the highest content among the elements. The ratio of Ag to S is almost 3:1, which means that the ratio of Ag_2_S to Ag must be 1:1. The Ag became decomplexed from dopamine as material properties such as plasmon absorbance and yellowness disappeared due to the changes in the size and structure. TEM (Figure 2B) also demonstrated that the average particle size of the AgNPs after reaction with S^2−^ grew from 10 nm to about 20–30 nm in diameter. This diminishing of the plasmon absorbance of AgNPs at 400 nm in the presence of S^2−^ against the blank was used as an analytical parameter for the determination of S^2−^. The working principle of a colorimetric assay of S^2−^ is schematically represented in Scheme 1. Figure 1A,B shows a typical UV-Vis absorption spectrum of the AgNP colloidal solution after reaction with S^2−^, and the relative spectrum change depended on the concentrations of S^2−^ in a range from 0.1 to 20 μM, obtaining a linear equation of A_400_ = 0.6828C_S_^2−^ − 0.0366 (R^2^ = 0.975) in a concentration range of 2–15 μM·S^2−^ with a detection limit of 0.03 μM, spanning two orders of magnitude. These results demonstrate that AgNPs are highly sensitive to S^2−^. In further studies, the effect of physiologically important species as potential interferents on the plasmon peak of AgNPs was examined and studied later.

### 3.2. Absorption and TEM Studies of Silver/Dopamine Nanoparticles after Incubation with Thiol Compounds

**Figure 3:**
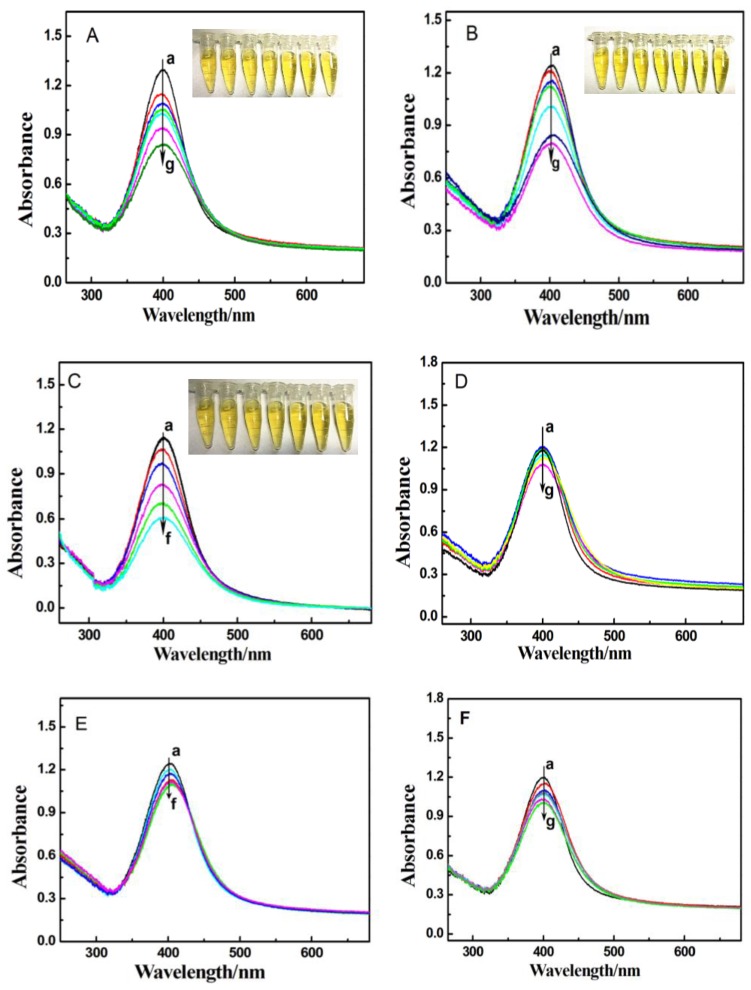
UV-Vis absorption responses of AgNPs in the absence (curve a) and presence of different concentrations of (**A**) Cys (from curve a to g: 0, 10, 15, 20, 30, 50, 100 μM), (**B**) Hcy (from curve a to g: 0, 10, 15, 20, 30, 50, 100 μM), (**C**) GSH (from curve a to f: 0, 10, 20, 30, 50, 100 μM), (**D**) GSSG(from curve a to g: 0, 10, 15, 20, 30, 50, 100 μM), and (**E**) MUA (from curve a to f: 0, 10, 20, 30, 50, 100 μM), and (**F**) N-cys (from curve a to g: 0, 10, 15, 20, 30, 50, 100 μM). The color changes of AgNPs before (the first tube) and after the reaction with different concentrations of (**A**) Cys (from curve a to g: 0, 10, 15, 20, 30, 50, 100 μM), (**B**) Hcy(from curve a to g: 0, 10, 15, 20, 30, 50, 100 μM), (**C**) GSH (from curve a to g: 0, 10, 15, 20, 30, 50, 100 μM) are shown in the corresponding inset. The reation time, 2 h.

It was found that thiol compounds have a strong effect on the plasmon resonance of AgNPs. Therefore, we studied the UV-Vis spectra of the AgNP plasmon in the presence of different thiol compounds including Cys, Hcy, GSH, GSSG, MUA, N-cys, and DTT. Figure 3 shows a typical UV-Vis absorption spectrum of AgNPs in the presence of different concentrations of Cys (Figure 3A), Hcy (Figure 3B), and GSH (Figure 3C). As can be seen, upon the addition of these reduced biothiols, the absorbance at maximum wavelength, 400 nm, decreased in different degrees, as shown in Figure 3. The decrease in plasmon intensity could be due to the interaction of the –SH of Cys, Hcy, and GSH with silver. Unlike these reduced biothiols, the molecule structure of MUA and N-cys, which also had a sulfhydryl group, did not lead to the diminishing of the plasmon absorbance of AgNPs (Figure 3D,F). In addition, oxidized biothiols GSSG, which had no free sulfhydryl group, did not induce a decrease in the plasmon absorbance of AgNPs (Figure 3E) or in MUA. According to TEM (Figure 2A), the average size of AgNPs formed through the reduction of AgNO_3_ with dopamine was approximately 10 nm and the particles were evenly distributed. The hydroxyl groups of dopamine were reported to be the main reducing groups for Ag+, and oxidation product polydopamine from this reaction process adsorbed the AgNP surfaces and stabilized the monodispersed AgNPs. However, the TEM images in Figure 5C,D further prove that, upon the addition of reduced biothiols, the size of AgNPs became larger with uneven distribution. These phenomena indicate that the AgNP structure broke down. Furthermore, as the incubating time with reduced biothiols increased (after about 24 h at room temperature), the AgNP colloidal solution severely broke down, and the as-formed complex between AgNPs and reduced biothiols was consequently precipitated from the dispersion, resulting in a clear and transparent dispersion, and small black particles gathered at the bottom of the tubes, as shown in Figure S2. From the above, although the addition of reduced biothiols including Cys, Hcy, and GSH to the AgNP colloidal solution had similar UV-Vis absorption responses as well as S^2−^, we can still discriminate S^2−^ from Cys, Hcy, or GSH based on the differentiation over the reaction time. In particular, the absorption responses for S^2−^ reached a constant value in only 20 min; for Cys, Hcy, or GSH, a constant value was not reached until after 24 h at room temperature. Thus, the differentiation of the absorption spectrum kinetics can be relied on to distinguish S^2−^ from Cys, Hcy, or GSH. The absorption spectrum change of AgNPs toward S^2−^ is compared with other thiol compounds in Figure S3.

**Figure 4:**
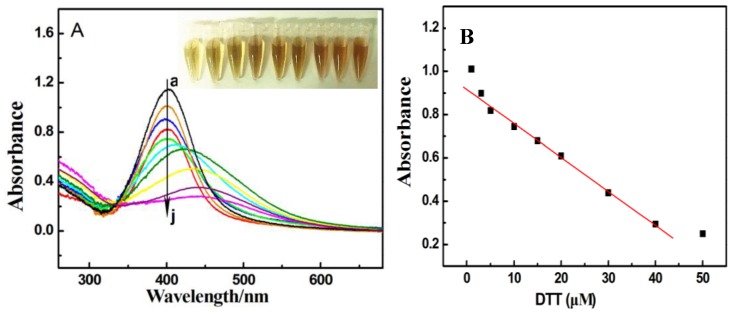
(**A**) UV-Vis absorption responses of AgNPs in the absence (curve a) and presence of different concentrations of DTT (from curve a to j: 0, 1, 3, 5, 10, 15, 20, 30, 40, 50 μM). The color changes of AgNPs before (the first tube) and after the reaction with different concentrations of captopril (from curve a to i: 0, 1, 3, 5, 10, 20, 30, 40, 50 μM) are shown in the corresponding inset. The reation time, 20 min. (**B**) UV-Vis absorption responses versus the DTT concentration.

**Figure 5:**
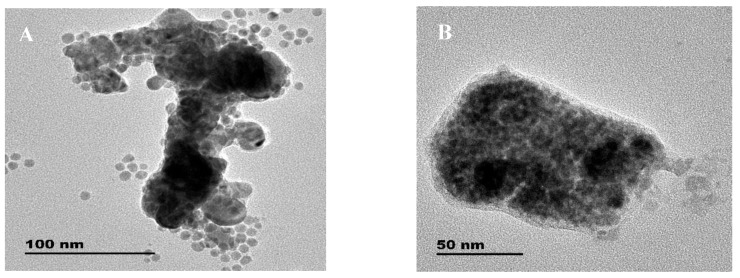

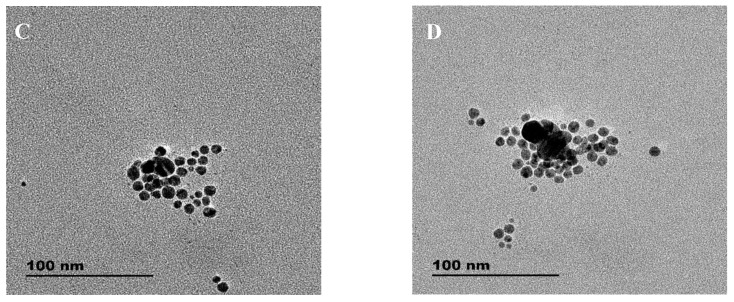
TEM image of dopamine-functionalized AgNPs in the presence of (**A**) and (**B**) 20 μM DTT, and (**C**) 50 μM Cys, (**D**) 50 μM GSH. The reation time for DTT, 20 min; The reation time for Cys and GSH, 2 h.

In addition, DTT, which had two proximal sulfhydryl groups, was chosen to examine its response after reaction with AgNPs. Figure 4 shows the absorption spectrum of AgNPs in the presence of different concentrations of DTT. As can be seen, when DTT was present in the detection system, the AgNP colloidal solution would change from bright yellow to reddish brown in 5 min (Figure 4A inset), implying the formation of aggregated AgNPs. Meanwhile, the absorbance peak of the AgNP colloidal solution at maximum wavelength 400 nm decreased and was red-shifted from 400 to 420 nm. These responses could be ascribed to the crosslinking interaction between the –SH of DTT and AgNPs. In particular, two proximal sulfhydryl groups of DTT can crosslink monodispersed AgNPs to form aggregated AgNPs with a larger particle size, resulting in the corresponding changes in color and absorbance peak. The aforementioned reaction mechanism is demonstrated in the TEM image. Figure 5A,B further proves that the size of the AgNPs changed and formed larger particles with uneven distribution after the addition of DTT. Different from the aforementioned reduced biothiols, DTT can significantly damage the structure of AgNPs and cause the formation of larger particles in 5 min with obvious color changes, while Cys, Hcy, and GSH needed more than 24 h to produce slight color changes. These characteristic spectrums of AgNPs in the presence of DTT actually form the basis for the development of an effective, direct, colorimetric method for the detection of DTT. Figure 4 shows typical absorption responses of AgNPs in the absence (Curve A) and presence of different concentrations of DTT, and the relative absorption intensity at 400 nm depended on concentrations of DTT in a range from 1 to 50 μM DTT, obtaining a linear equation of A_400_ = 0.9159 − 0.0157C_DTT_ (R^2^ = 0.997) in a concentration range of 3–40 μM DTT with a detection limit of 0.3 μM.

### 3.3. Interference Study

**Figure 6:**
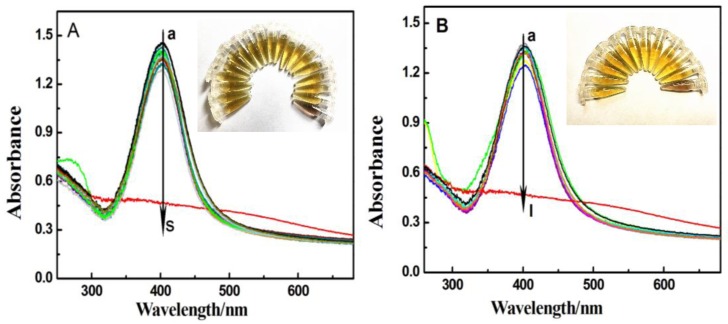
(**A**) The color changes and UV-Vis absorption responses of AgNPs in the absence (curve a) and presence of different species (from curve b to t: tryptophan, histidine, proline, alanine, lysine, phenylalanine, leucine, threonine, arginine, aspartic acid, glycine, valine, serine, glutamine, methionine, tyrosine, 40 μM; 0.01% BSA and S^2−^, 20 μM ). (**B**) The color changes and UV-Vis absorption responses of AgNPs in the absence (curve a) and presence of different species (from curve b to l: glucose, glutamic acid, 5-hydroxytryptamine, uric acid, lactate, ATP, norepinephrine, hypoxanthine, H_2_O_2_, and sodium ascorbate, 20 μM and S^2−^, 20 μM ). The reation time for S^2−^ and DTT, 20 min. The reation time for other species, 2 h.

To investigate the selectivity of silver/dopamine nanoparticles, potential interferences such as other amino acids, various cations, anions, and physiologically important species under physiological conditions were studied. Each kind of species was examined in parallel under the same conditions as those of S^2−^ and thiol compounds. As shown in Figure 6, the separate addition of other amino acids and physiologically important species exceeding the upper limit of the physiological concentration range to the AgNP colloidal solution caused minor absorption spectrum changes in the AgNPs at 400 nm but no color change. As compared with those of the aggregation triggered by S^2−^ and DTT, these results substantially suggest that these amino acids and physiologically important species did not interfere with the S^2−^ and DTT sensing.

**Figure 7:**
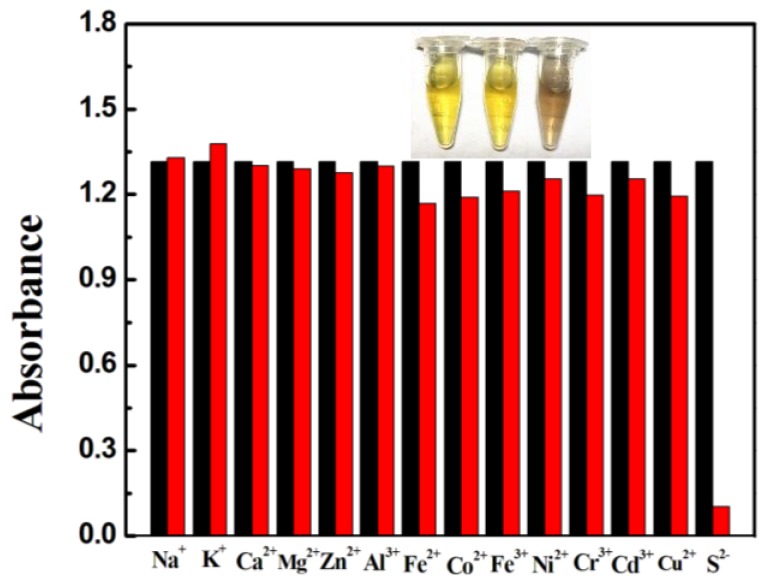
UV-Vis absorption responses A_400_ of AgNPs in the absence (black bars) and presence of various metal ions containing 50 μM EDTA. The concentration of Fe^3+^, Fe^2+^, Zn^2+^, Cu^2+^, S^2−^, Al^3+^, 20 μM; Cr^3+^, Cd^3^, Co^2+^,5 μM; Mg^2+^, Na^+^, K^+^, Ca^2+^, 50 μM; Photographs from curve left to right: AgNPs with 80 μM EDTA, AgNPs with 20 μM Cu^2+^ and 80 μM EDTA, AgNPs with 20 μM Cu^2+^. The reation time for metal ions, 2 h.

Ma et al. [36] has demonstrated that silver/dopamine nanoparticles have high selectivity and sensitivity towards Cu^2+^ based on the coordination ability between Cu^2+^ and the nitrogen and oxygen atoms of dopamine without interference from other metal ions, indicating interference from Cu^2+^ in our work for the selective detection of sulfide and DTT. Herein, we once again examined the absorption responses of AgNPs toward 12 other kinds of conventional metal ions: Fe^3+^, Fe^2+^, Zn^2+^, Al^3+^, Cr^3+^, Cd^3^, Co^2+^, Mg^2+^, Na^+^, K^+^, Ca^2+^, and Cu^2+^. The results suggest that these cations caused minor absorption responses changes, except for Cu^2+^ Fe^3+^, Fe^2+^, Cd^3+^, and Co^2+^, which were considered as the main interfering species. However, we can chelate and shelter the interference cations by the addition of ethylenediaminetetraacetic acid (EDTA). As shown in the red columns of Figure 7, if EDTA coexisted with these cations, the AgNP colloidal solution remained yellow (Figure 1B), and there is no change in absorption response because these interference ions were chelated by EDTA, which prevented the binding reaction of these cations and dopamine. The inset of Figure 7 showed the color change for silver/dopamine nanoparticles and Cu^2+^ in the absence and presence of EDTA. As can be seen, when EDTA coexisted with Cu^2+^, AgNPs displayed a bright yellow color just as in the absence of Cu^2+^. Thus, by using the chelating reagent EDTA, the colorimetric sensor based on AgNPs has excellent selectivity to sulfide and DTT over other coexisting metal ions.

Considering that it is quite simple for Ag^+^ to form the precipitates from the dispersion when the reaction between Ag^+^ and many anions occurs, such as Cl^−^ for AgCl and SO_4_^2−^ for Ag_2_SO_4_,, the selectivity of AgNPs for sensing S^2−^ and DTT was thus evaluated by comparing their absorption response changes toward those of 15 other kinds of conventional anions, Cl^−^, F^−^, Br^−^, I^−^, SO_4_^2−^, SO_3_^2−^, ClO_4_^−^, NO_3_^−^, CH_3_CO_2_^−^, CO_3_^2−^, ATP, AMP, ADP, PPi, and PO_4_^3−^, with different concentrations at the physiological level. As shown in Figure S4, these anions induced negligible plasmon absorbance changes in AgNPs at 400 nm, implying a stronger coordination effect between dopamine and AgNPs than the associated interaction between Ag^+^ and the tested anions. The aforementioned results sufficiently suggest that AgNPs have a high selectivity for S^2−^ and DTT, allowing potential applications in complex samples.

### 3.4. The Determination of S^2−^ in Fetal Bovine Serum

To validate the performance of the developed colorimetric detection method in real samples, we then applied as-synthetized AgNPs to directly detect the sulfide concentration in fetal bovine serum. The treated serum as described in the experimental section was added into the aqueous dispersion of AgNPs. The average sulfide concentration in three replicate samples was about 16.3 μM, which was consistent with other reported results about sulfide concentrations in serum [39,40]. For the spiked-recovery assays, the results revealed that the recoveries of the added S^2−^ with the known concentration 5, 10, 20, 30, and 50 μM were 97%, 99%, 103%, 98%, and 99%, respectively, which was acceptable, indicating that it can be used to monitor sulfide in real biological samples including bioassays, nanotechnology, and clinical diagnostics.

## 4. Conclusions

A facile, highly selective and reliable new method for the determination of sulfide and 1,4-dithiothreitol (DTT) are described herein. The method is based on the in situ formation of silver nanoparticles using dopamine as a reducing and stabilization agent, which showed corresponding changes in the color and the absorbance peak toward sulfide and 1,4-dithiothreitol (DTT). Field transmission electron microscope (TEM) images further proved that the size and the morphology of AgNPs changed, and formed larger particles with uneven distribution after the addition of sulfide and DTT. In conclusion, this measurement based on the in situ formation of AgNPs simplifies the operation and is completely economical and eco-friendly. Benefiting from the unique optical properties of AgNPs, the determination of sulfide and DTT can be accomplished in 10 min and we can achieve the determination of sulfide and DTT with the naked eye, which is greatly suitable for the analysis of sulfide and DTT in fieldwork. The sensor utilizing as-formed AgNPs is promising for the further establishment of microfluidic paper-based analytical devices for point-of-use diagnostics, without external power supplies or supporting equipment based on the color change of AgNPs.

## Supplementary Materials

The following are available online at www.mdpi.com/1424-8220/17/3/626/s1, Figures S1–S4.
