# Supplementary material for: A Colorimetric Sensor for the Highly Selective Detection of Sulfide and 1,4-Dithiothreitol Based on the In Situ Formation of Silver Nanoparticles Using Dopamine
Source: Sensors (Basel). 2017 Mar 20;17(3):626. doi: 10.3390/s17030626 (PMC5375912; doi:10.3390/s17030626)
Supplement: Supplementary file 1 [file sensors-17-00626-s001.pdf]

Supplementary Materials: A Colorimetric Sensor for the Highly Selective Detection of Sulfide and 1,4-Dithiothreitol Based on the *In Situ* Formation of Silver Nanoparticles Using Dopamine

Lingzhi Zhao [1,2,*], Liu Zhao [3], Yanqing Miao [1], Chunye Liu [1] and Chenxiao Zhang [2]

[1] Department of pharmacy, Xi'an Medical College, Xi'an 710021, China; miaoyanqing2006@163.com (Y.M.); doris8976@163.com (C.L.)
[2] Laboratory of Analytical Chemistry for Life Science of Shaanxi Province, School of Chemistry and Chemical Engineering, Shaanxi Normal University, Xi'an 710062, China; cxzhang@snnu.edu.cn
[3] Beijing Research Center of Agricultural Standards and Testing, Beijing 100097, China; zhaoliu@nercita.org.cn
* Correspondence: oldskyhappy_zlz@163.com; Tel.: +86-158-2958-3060

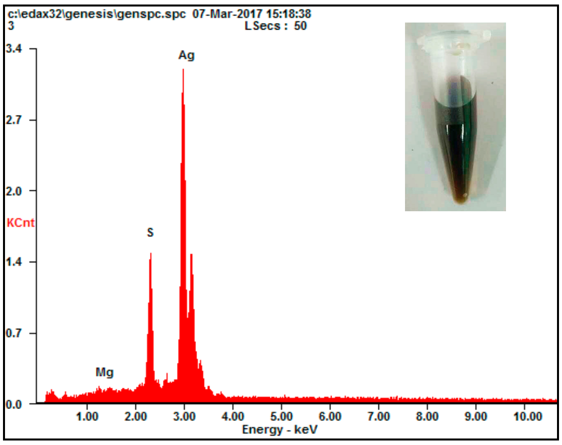

Element	Wt %	At %
MgK	00.92	03.11
S K	11.81	30.30
AgL	87.27	66.58

Figure S1. Energy dispersive X-ray spectroscope (EDS) spectrum and photo of the mixture of 5 mM $AgNO_3$ and 5 mM Na_2S.

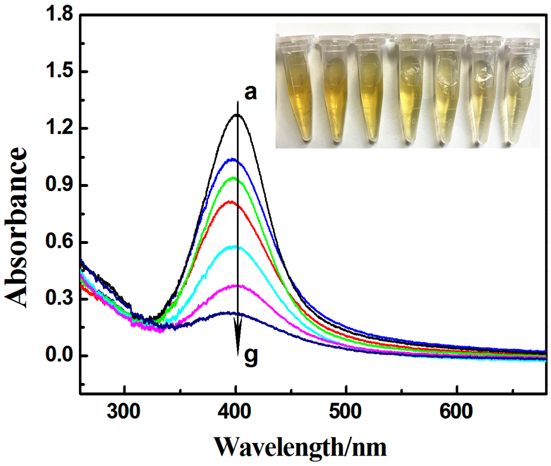

Figure S2. UV-Vis absorption responses of AgNPs in the absence (curve a) and presence of different concentrations of GSH (from curve a to g: 0, 10, 15, 20, 30, 50, 100 μM). The color changes of AgNPs before (the first tube) and after the reaction with different concentrations of GSH (from curve a to h: 0, 10, 15, 20, 30, 50,100 μM) for 24 h later.

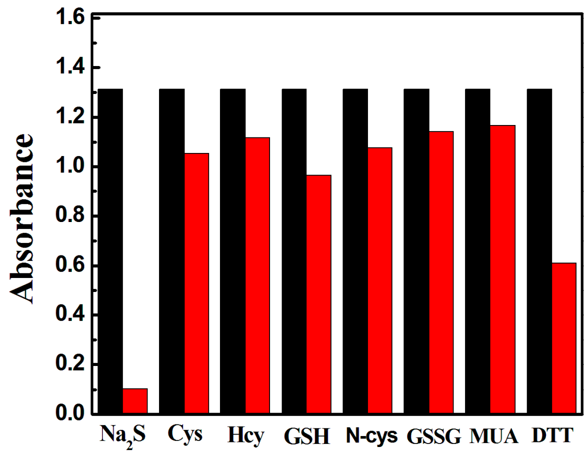

Figure S3. UV-Vis absorption responses of AgNPs in the absence (black columns) and presence of different thiol compounds (red columns). The reation time for S^{2-} and DTT, 20 min. The reation time for Cys, Hcy, GSH, GSSG, MUA N-cys, 2 h.

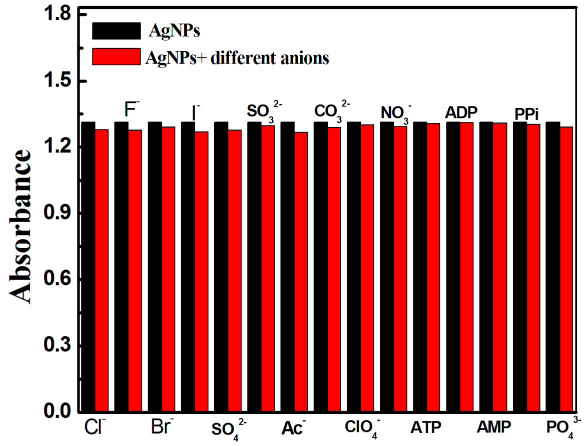

Figure S4. UV-Vis absorption responses of AgNPs in the absence (black columns) and presence of various anions (red columns) in aqueous solution. The concentration of Cl^-, NO_3^-, PO_4^{3-}, SO_4^{2-}, 100 μM; ATP, AMP, ADP, PPi, ClO_4^-, $CH_3CO_2^-$, CO_3^{2-}, SO_3^{2-}, 50 μM; F^-, Br^-, I^-, 20 μM.
